# PhotoElasticFinger: Robot Tactile Fingertip Based on Photoelastic Effect

**DOI:** 10.3390/s22186807

**Published:** 2022-09-08

**Authors:** Dinmukhammed Mukashev, Nurdaulet Zhuzbay, Ainur Koshkinbayeva, Bakhtiyar Orazbayev, Zhanat Kappassov

**Affiliations:** 1 Institute of Smart Systems and Artificial Intelligence, Nur-Sultan 010000, Kazakhstan; 2 Robotics Department, Nazarbayev University, Nur-Sultan 010000, Kazakhstan; 3 Physics Department, Nazarbayev University, Nur-Sultan 010000, Kazakhstan

**Keywords:** optical sensor, tactile sensing, photoelastic effect

## Abstract

The sense of touch is fundamental for a one-to-one mapping between the environment and a robot that physically interacts with the environment. Herein, we describe a tactile fingertip design that can robustly detect interaction forces given data collected from a camera. This design is based on the photoelastic effect observed in silicone matter. Under the force applied to the silicone rubber, owing to the stress-induced birefringence, the light propagating within the silicone rubber is subjected to the angular phase shift, where the latter is proportional to the increase in the image brightness in the camera frames. We present the calibration and test results of the photoelastic sensor design on a bench using a robot arm and with a certified industrial force torque sensor. We also discuss the applications of this sensor design and its potential relationship with human mechano-transduction receptors. We achieved a force sensing range of up to 8 N with a force resolution of around 0.5 N. The photoelastic tactile fingertip is suitable for robot grasping and might lead to further progress in robust tactile sensing.

## 1. Introduction

Physical interaction with the environment is always accompanied by mechanical stimuli, for which the human body is endowed with mechanisms to sense it. Peripheral terminals of the human somatosensory system transduce sensory stimuli into signals that propagate to the central nervous system [1]. Transduction mechanisms, specifically mechano-transduction of the skin, are thought to be the hidden ingredient of human dexterity in object manipulation since they provide an essential state of physical contact when we explore and manipulate objects [2]. Since the introduction of industrial robots within a car assembly line in General Motors Company, tremendous effort was made to endow the robot arms with sensing capabilities similar to the aforementioned mechanoreceptors [3,4]. Various transduction methods, aiming at crafts on the mechano-transduction system in the human skin were proposed to meet the more and more puzzling tradeoffs and constraints on the design of tactile sensors for robots [5]. Electrical transduction, that present in piezoelectric resistors [6], transistors [7], capacitors [8], micro electro-mechanical systems [9], electro-magnetic [10,11] has been, historically, the most commonly exploited method for contact force sensing. In these sensors, physical contact modulates electrical signals. Degradation of sensing capabilities with time, susceptibility to external electromagnetic interference are the most common drawbacks of them.

On the other hand, research in robot manipulation anticipated the need for optical tactile sensors that are more robust and indifferent to the external electromagnetic fields [12,13]. This initiated various transduction methods concerned with the use of light propagation phenomenons, including refraction, reflection, diffraction, interference, etc. Usually, these sensing systems based on optical transduction are built upon image sensors, basically RGB cameras, and silicone rubbers, e.g., [14].

Mechanical properties of the aforementioned sensors, share to some extent similarities with other types of sensors, which are based on different transduction methods but that use silicone rubber, for example, hall effect sensors to detect the magnetic field generated by magnets embedded within a rubber [8,10,15,16] and capacitance sensing based on dielectric properties of silicone rubber [17].

Several works employed fine optical structures in tactile robotics. Authors of [18] presented a photonic tactile sensor based on polymer material for multi-point contact with force detection range of 0–3 N at 1 Hz. Another photonic sensor [19] made of polyurethane-silicone rubber was designed for a soft robotic hand. A vision camera was used to detect light reflected from a thin silver-coated surface of a transparent silicone [20]. An event-based camera used to detect movements of reflective markers embedded in opaque rubber [21]. Infrared sensing was applied to detect the light reflected from the contact surface between the silicone rubber and an object [22].

Due to a relatively high density of transducers in RGB cameras, designers were competing to achieve higher spatial resolution by manufacturing thin and malleable reflective surfaces to capture small geometrical features of contact patterns [21,23,24,25]. However, the multilayer structure and the reflective markers make the manufacturing process complex, e.g., masking a multilayer structure [26], or sometimes requiring advanced manufacturing devices, e.g., thin film magnetron physical vapor deposition [27]. For the aforementioned optical tactile sensors, the complex fabrication process can be considered as the drawback, which can be solved by *photoelastic* optical tactile sensors discussed in the following.

Bertholds et al. [28] were among the first who applied the photoelastic effect as a tool for mechanical pressure sensing. Bertholds et al. used one single-mode optical fiber (126 μm), pair of polarizers, a laser, and two photo-detectors.

With time, the use of the photoelastic effect evolved into tactile sensing technology. Several normal, shear, and torque sensors working on the photoelastic effect were developed. Dynamic tactile sensor by the authors of [29] could measure the normal force and even detect object slippage. The authors used a high radiance emitter and PIN photodiode as a receiver. The output signals from the optical filter were used to detect both slippage and normal forces. Their sensor was capable of measuring the normal force up to 1.7 N. At the same time, the slip mechanism could measure the slip rates as 0.1 mm/s. The applied normal force could be estimated by analyzing the fringes formed when photoelastic material was subjected to mechanical stress under white light. These fringes were viewed via a polariscope. The authors of [30] used this technique to estimate the load on photoelastic material, whereas the authors of [31] performed torque measurements. In the former work, the authors reported that the maximum fringe order of their photoelastic material is equivalent to 95 N of normal load applied to it. They used neural networks to correlate the normal load with the fringe order. A low error rate of 2.78% was achieved with a 1 M-pixel resolution camera. Similarly, the latter authors [31] applied a neural net to analyse camera images in a torque estimation task. The authors reported a dynamic range of 7.9 to 16.9 Nm and the error rate of 0.4%. Some approaches involve microscopic measurements, for example, authors of [32] created a metamaterial structure by mixing granular photoelastic particles to estimate both the direction and amplitude of applied force.

The authors of [33] developed the photoelasticity-based approach to analyze foot images in the whole-field sensing applications for diabetic people. In contrast to the aforementioned approach, photoelasticity could also be applied in haptics. For example, the authors of [34] developed a touch panel based on the photoelastic effect. The authors showed that the detected pressure can be used for scrolling the mouse in a personal computer.

In this paper, we focus on straightforward manufacturing process and durability rather than the detection of geometrical features on the sensing surface. We conjectured that a standard camera-based tactile sensing system [12] built of transparent silicone rubber and light source in combination with two linear light polarizers would be sufficient for tactile contact sensing. The sensor works as follows. The orientation of the first polarizer is set orthogonal with respect to the second one so that the light does not pass through at resting state of the sensor. As the sensor is engaged in tactile contact, the silicone layer deforms under pressure at the point of contact proportionally to the force of pressure. The stress and strain in this rubber can be visualized due to the photoelastic effect. We then leverage this photoelastic effect in the rubber to detect the applied force.

The contribution of this work is a tactile sensing approach in reference to the photoelastic mechano-transduction [35,36,37,38]. Similar to human mechanoreceptors, photoelastic mechanoreceptors respond to mechanical stimuli, such as pressing. To reinforce this approach, we constructed a conceptual apparatus featuring a photoelastic effect. The apparatus is referred to as a PhotoElasticFinger. To perform quantitative benchmark and qualitative efficacy verification, we equipped a gripper of a torque-controlled robot arm with PhotoElasticFinger.

**Figure 1:**
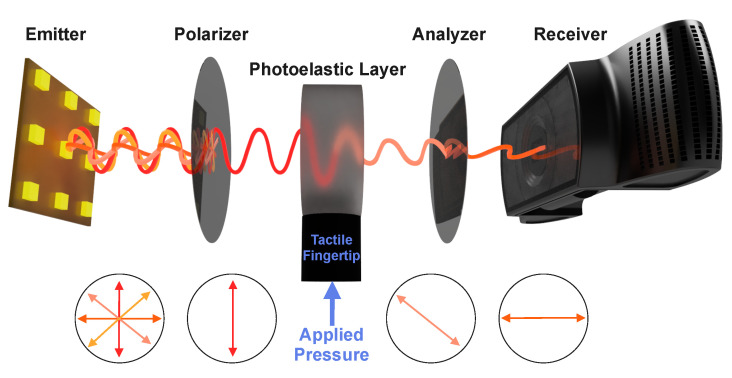
Illustration of the proposed photoelastic sensor for the PhotoElasticFinger. Red and orange beams represent unpolarized light from the array of LEDs. After passing through the polarizer, the polarized light enters the silicone under pressure, where the angular phase shift is changed due to stress-induced birefringence. The shifted light passes through the analyzer. The more pressure is applied, the more phase-shifted light passes through the analyzer.

The novelty of our approach is the structure of the tactile sensor. We combined a photoelastic silicone rubber with opaque silicone rubber. The hardness of the rubbers are different. Photoelastic rubber is softer than opaque rubber. The hard rubber is designed as tactile fingertip (Figure 1) that interacts with the environment. The soft rubber (Photoelastic Layer in the same figure) deforms due to the applied pressure during the physical interaction. Any deformation in the photoelastic layer modifies its refractive index. A numerical simulation of the distribution of the refractive index change has been validated by calibration of the photoelastic sensor.

The rest of the letter is structured in the following way: In Section 2, we describe the concept behind the phenomena, as well as the manufacturing process with experimental setup. The discussion of the results found during the manufacturing of the sensor with its calibration are given in Section 3. Finally, in Section 4, we conclude the work.

## 2. Principle and Methodology

The concept of the proposed photoelastic sensor is illustrated in Figure 1. The light of light emitting diodes (LED) is filtered by a polarizer and detected by a camera with analyzer placed in front of the lens of the camera. The photoelastic sensor is placed between the polarizer and analyzer.

The photoelastic effect and its numerical simulation for the photoelastic layer are described first. More details on modeling the light propagation can be found elsewhere, e.g., [37]. The fabrication process of the photoelastic layer merged with the tactile fingertip is described next; and it is followed by the description of a robot platform to which the sensor was attached.

### 2.1. Photoelasticity for Sensing

To measure the stress-induced birefringence (dependence of refractive index on the polarization of propagating light) a typical setup of two polarizers is employed. The light emitted from an array of LEDs is prepolarized vertically by the polarizer. The polarized light then propagates through a compressible transparent layer, whose refractive index depends on the applied pressure. The beam’s polarization is then analyzed by recording the light intensity transmitted through analyzer by using a photosensitive matrix (a photosensor of a web camera). Since the light intensity function has a higher quality factor at minima, the polarizer and analyzer have an orthogonal orientation. All the details of photoelasticity theory are described in Appendix A.1. In this work, for the sake of simplicity, we focus on the relative change of the light intensity and consider only linearly polarized light beams.

To demonstrate the working mechanism of the stress-induced angular phase shift and its applicability to a sensor, the numerical simulations were performed using a commercial numerical solver COMSOL Multiphysics^™^. All the details of the numerical calculations are described in Appendix A.2.

**Figure 2:**
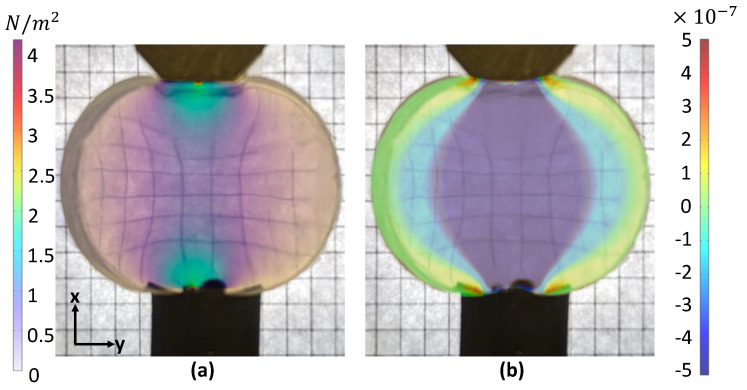
Semitransparent color−maps simulated in COMSOL Multiphysics^™^ laid over the picture of silicone under pressure. (**a**) Von Misses Stress |σ| distribution map. (**b**) Color map distribution of the refractive index change $n_{x}-n_{y}$. The checkered pattern on the background used to highlight the deformation of silicone layer.

**Figure A1:**
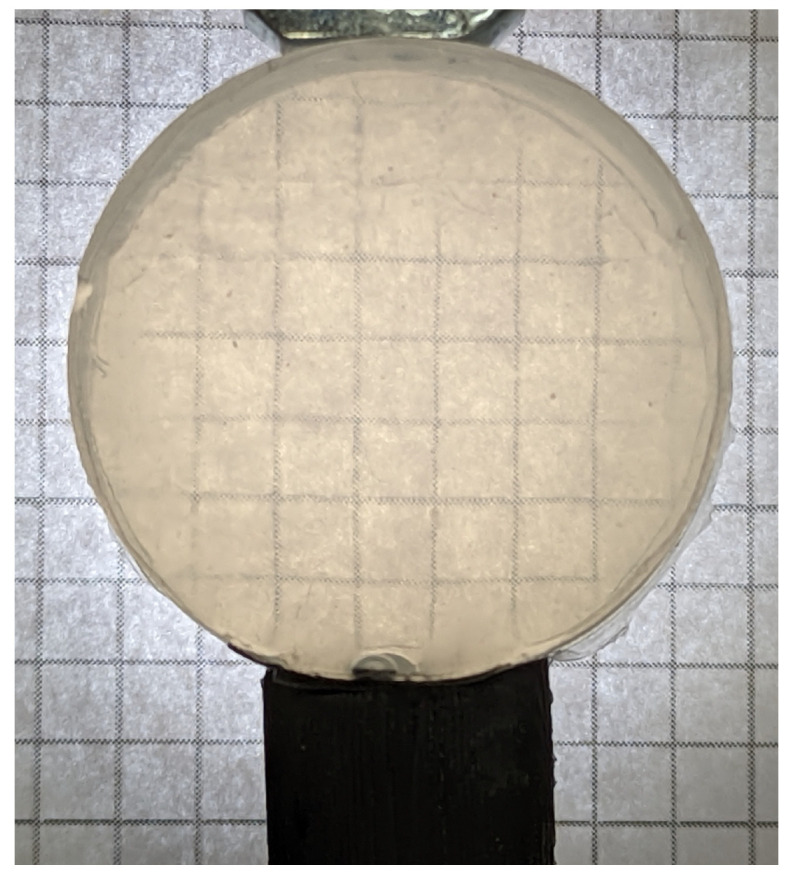
Photoelastic layer made from two component silicone rubber at resting state. There is no deformation before pressure was applied.

The obtained numerical results are shown in Figure 2a, where the calculated colormap distribution of the von Mises stress is overlapped over the photograph of a real deformation of the silicone layer. The original photograph of the silicone layer without deformations is illustrated in Figure A1. From Figure 2a, a higher amount of stress is located on the smaller contact areas due to a higher pressure, since pressure p=F/A, where *F* and *A* are the force and contact area, respectively. The corresponding relative change in the refractive index $n_{x}-n_{y}$ is plotted in Figure 2b and laid over the deformed silicone. It can be observed that the highest $n_{x}-n_{y}$ occurs in the middle of the silicone, where the most stress is observed. From the calculated $n_{x}-n_{y}\approx 5\times 10^{-7}$ the relative angular phase shift $\Delta_{xy}\approx 0.05$ and the relative change in the light intensity $sin^{2}\left(\frac{\Delta_{xy}}{2}\right)\approx 6.3\times 10^{-4}$ (see Appendix A.1). Such a small change in the intensity is still well above the noise levels of commercial consumer-grade photosensors [39], allowing the use of cheap photosensors to build the photoelastic sensors.

### 2.2. Design and Fabrication

**Figure 3:**
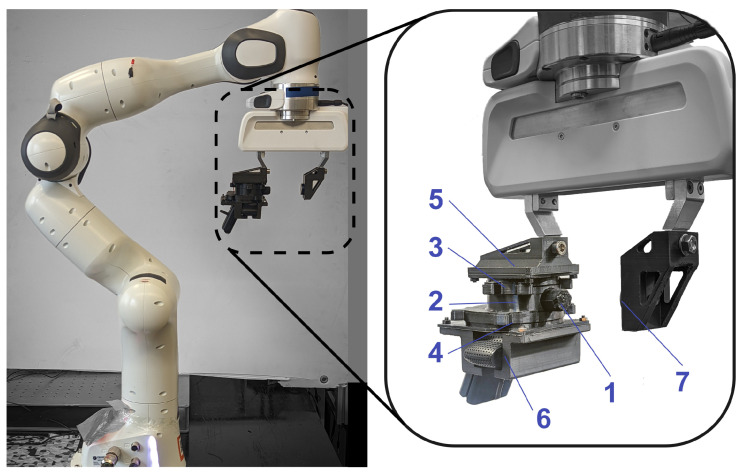
Test bench with PhotoElasticFinger: 1—sensing tip (tough silicone rubber), 2—transparent photoelastic layer (soft silicone rubber), 3 and 4—a pair of polarizers with orthogonal polarization planes, 5—light source, 6—RGB camera, and 7—calibration device (WITTENSTEIN force/torque sensor). The exploded view of the sensor is provided in Appendix B Figure A3 and in Supplementary Materials.

To create the stress (Figure 2) on the optical part of the photoelastic sensor, a fingertip made from a silicone rubber part (element ‘1’ in Figure 3) was attached to the photoelastic part. To maximize the stress in the central area and increase the overall sensitivity of the sensor, the fingertip’s shape was chosen to be cylindrical, with a small part protruding into the photoelastic silicone layer.

The photoelastic layer was formed by molds manufactured using a rapid prototyping printer based on fused deposition modeling (Ultimaker Cura S5, PLA filament). To avoid light reflection on the sensing rubber’s boundaries and ensure the sensor’s optimal performance, it is vital to have smooth surfaces of the photoelastic layer. To this end, a gasket (thin layer of the glassy film) was placed between the 3D printed mold and the transparent sides of the silicone. Such a fabrication process allows the creation of a transparent photoelastic layer with smooth surfaces.

A two-component silicone resin was used to fabricate the sensing and pressing parts. For the transparent photoelastic part of the sensor, we used softer silicone (Sorta Clear 12, Smooth-on, Young’s modulus E=0.4MPa and Poisson’s ratio v≃0.38). The softness of the silicone parts was controlled by the ratio of mixing components (A and B), which was found to be optimal at 1:1. To ensure effective transfer of the force vector on the photoelastic layer, the fingertip was made from a more rigid two-component resin (Smooth-On, Sorta Clear 40), mixed in the 1:10 ratio. Moreover, to isolate the photoelastic layer from the stray light, the fingertip was made opaque by adding black pigment (Smooth-On Silc Pig Silicone Color Pigments). The silicone sensing layer’s overall dimensions are 15 mm in height and 35 mm in diameter). The adjacent fingertip is 17 mm in length and 15 mm in diameter (see Figure A3). To exploit all LEDs at full while keeping a compact design, we used polarization filters with a diameter of 50 mm.

Finally, the photoelastic layer, two polarizers, and a camera were placed into the modular enclosure, which was fabricated using the same material used in mold manufacturing. To achieve fast interchangeability of the silicone matter and to facilitate the assembly/disassembly of the sensor, strong magnets were used to attach all parts of the sensor.

### 2.3. System Description

**Figure 4:**
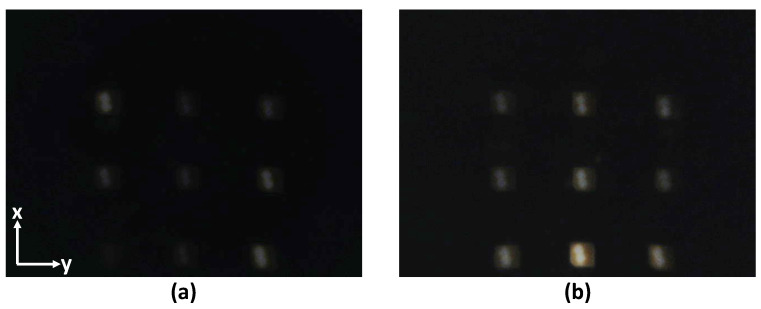
Two samples from the camera views: (**a**) resting state (average intensity = 5.99, the amplitude of applied force is zero) and (**b**) deformed state (average intensity = 13.45, the amplitude of the applied force is 7.00 N. (In these images, the overall intensity is increased by 10% for clarity.)

A 920 × 1080 pixels consumer-grade web camera (Logitech C920, Switzerland) was used to observe the photoelastic effect by capturing the change of the intensity of light at 30 frames per second. To keep the frame rate in this range, the exposition of the camera was manually set to a low level (200 in the range between 3 to 2047 in the camera software), which prevents the saturation of the camera sensor’s by the light. The camera’s focus was also set to the minimum focus distance to ensure that the LED array stays focused (the camera’s focal length is only 3.67 mm, whereas the distance between the camera lens and the printed circuit board (PCB) of the LEDs array is 40 mm). The camera’s diagonal field of view (FOV) is $78^{\circ }$, enough to capture all 9 LED lights in proximity. Examples of the snapshots taken by the camera capturing the light from this array are shown in Figure 4.

The LEDs are connected in series. The light intensity was controlled by the current that we supply to our custom-made 3 × 3 array of LEDs (high power 5050 lighting diode, Seoul semiconductor, Korea), with the supplied voltage of 14.5 V and a current of 100 mA. As a result, diodes emitted light with the intensity equivalent luminance of 180 Lux, which was measured using a Digital Lux Meter (LX1010B, Alion, Kowloon, Hong Kong). A denser array of LEDs was used instead of a single LED to increase the uniformity of the light intensity and reduce the effect of the diffraction inside the photoelastic layer. In addition, distributed high-power LEDs are more convenient for heating power management.

**Figure 5:**
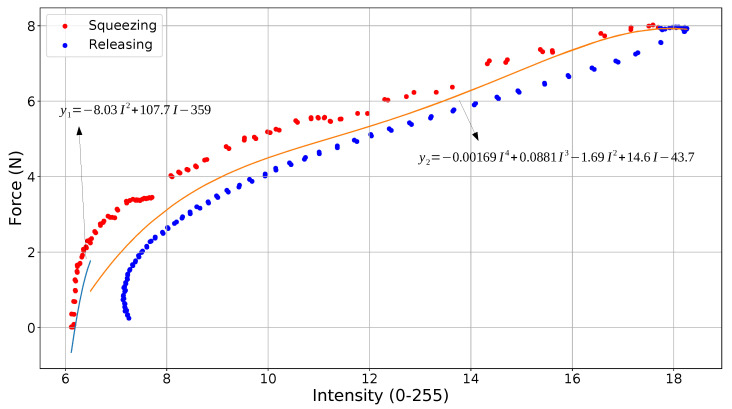
Light intensity variation due to the applied force on photoelastic silicone rubber in the proposed sensor design.

A 6-axis force/torque sensor (HEX 21, Wittenstein, Igersheim, Germany) with an accuracy of 1% was used as ground truth (label 7 in Figure 3) to calibrate the PhotoElasticFinger and plot the relationship between light intensity and force. Figure 5 illustrates the calibration plot. The ground-truth force sensor that provides actual force was mounted on the second finger of a robot gripper. The two sensors were facing each other such that geometrical middle points of the sensing surfaces were coincident. The force/torque sensor was sampled at the rate of 500 Hz and resolution of 10 Bits.

All of the components of the sensor discussed so far were attached onto a 7-DOF robot platform (Franka Emika Panda) for calibration, see Figure 3. The robot manipulator equipped with two finger gripper on the end-effector was used. The fingertip of our sensor was used as an interface between the objects to be manipulated and the robot. The sensor was installed onto one of the grippers, as it is shown in Figure 3. Specifically, we controlled the position and velocity of the gripper using *‘actionlib’*, and we recorded the data using *‘rosbag’* tools in ROS.

## 3. Results and Discussions

In this section, we describe the calibration of the photoelastic layer and discuss its force sensing characteristics when the sensor is attached to the robot end-effector.

### 3.1. Calibration

The PhotoElasticFinger was calibrated to estimate the normal force in SI units. Since our sensor is made of soft silicone resin, hysteresis is unavoidable in rubber-like material, since they act as mechanical low-pass filters [40]. The measurements of the PhotoElasticFinger sensor are affected by the previous stress applied. Designating that, it takes some time for silicone to recover to its previous state. Consequently, the presence of hysteresis appears to be the main source of error in photoelastic soft tactile sensors.

To decrease the effect of hysteresis and find a fit line, we performed measurements quasi-statically [41].

**Figure A2:**
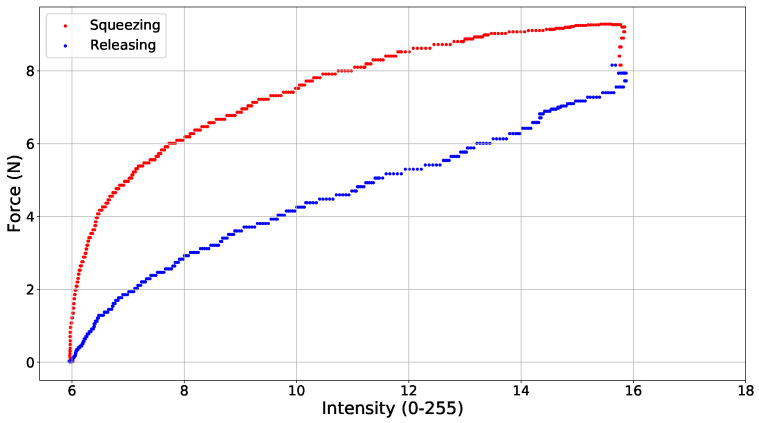
The calibration graph without a quasi-static regime.

**Figure A3:**
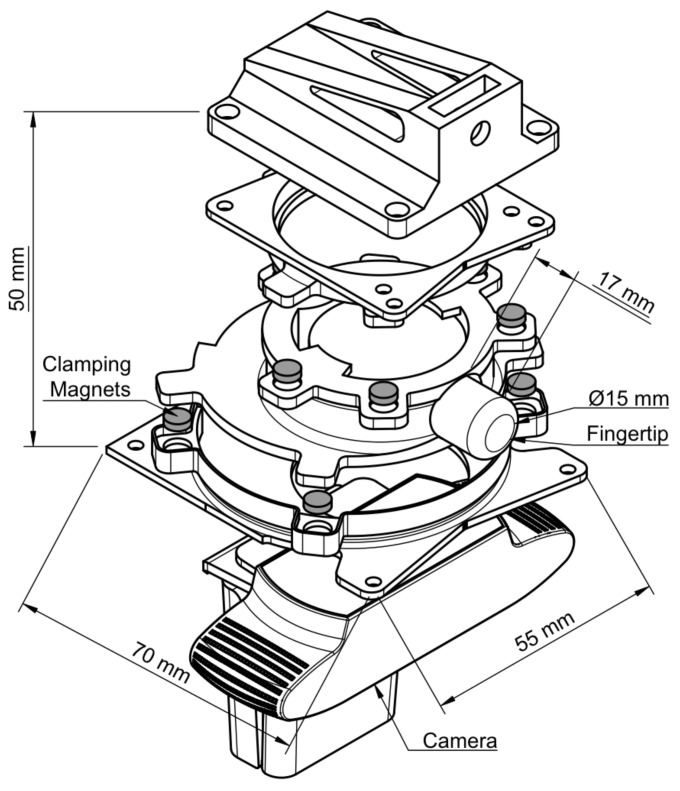
Robot Gripper and the exploded view of the modular sensor enclosure and photoelastic layer.

Figure 5 shows the force versus light intensity graph for squeezing and releasing the sensor. Loading and unloading the photoelastic silicone rubber are depicted in red and blue, respectively. Each measurement point in the plot is the average of 10 s measurements. In total, there were 100 points at which the silicone rubber was kept squeezed (1000 s or 16.6 min). In this connection, the shape of the rubber was not returned to its resting state by the end of the experiment, which resulted in the difference between the initial (red) and final (blue) light intensity values. This is known as compression set testing, by which the shape memory effect of the rubber can be quantified, as it takes time for the rubber to return to its original shape after prolonged compression. We did not observe this difference of the light intensity values when calibration was performed within 5 s of loading and 5 s of unloading (see Figure A2). The hysteresis curve is higher for the more dynamic deformation ($\frac{5\;\mathrm{mm}}{5\;s}$) than for the quasi-static deformation ($\frac{5\;\mathrm{mm}}{8\;\min}$).

To map the light intensity values captured by the camera into the force measurements, we implemented a piece-wise approximation function, which is drawn on the average fitting line between loading and unloading curves. The piece-wise consists of two parts, rising non-linear, and constant linear parts. The first part of the piece-wise function is used to estimate the forces up to 2 N. The second part of the piece-wise approximation function is derived for higher forces between 2 N and 8 N.

### 3.2. Force Sensing

To verify this approximation function, which to some extent disregards the real hysteresis of the silicone rubber, we performed several experiments that involve dynamic change of the contact force. We quantify the negative effect of hysteresis with Root-Mean-Square Error (RMSE) between the estimated and actual forces.

**Figure 6:**
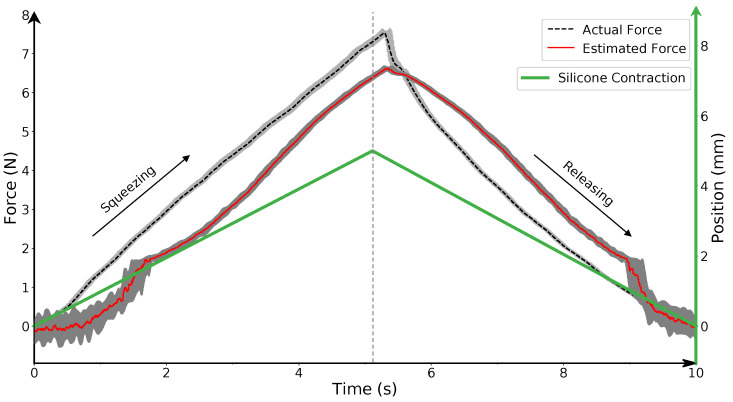
Comparative force sensing performance of the PhotoElasticFinger. Actual force is provided by an industrial force-torque sensor and compared with the estimated force provided by the proposed PhotoElasticFinger sensor. The gray areas represent the variance for 10 trials. Right-hand axis (green) represents the position of the gripper. Gripper speed = 1 mm/s.

Figure 6 illustrates the comparative performance in force estimation for the developed PhotoElasticFinger sensor when the gripper speed was 1 mm/s (10 trials). The data from the force-torque sensor (Wittenstein) and from the PhotoElasticFinger sensor were collected simultaneously using *‘rosbag’* service in ROS. Gray areas in the graph represent the deviation of the forces from both sensors. The position of the gripper (green line) is shown in the same time scale as the Cartesian distance units shown on the right-hand side axes (green). One can observe two features. First, there is higher variance in estimated force for the small displacement (0–2 mm) than for the maximum (5 mm) compression. Second, a constant bias between the actual and estimated force is present. Whereas the first feature is caused by the sensitivity during the absence of light (Figure 4a) and the noise present in the camera, which is amplified by the stray light from the ambient, the second feature is conditioned by our neglection of the hysteresis. However, this is a reasonable trade-off since the RMSE is only 0.91 N. This was derived for the gripper velocity of 1 mm/s.

**Figure 7:**
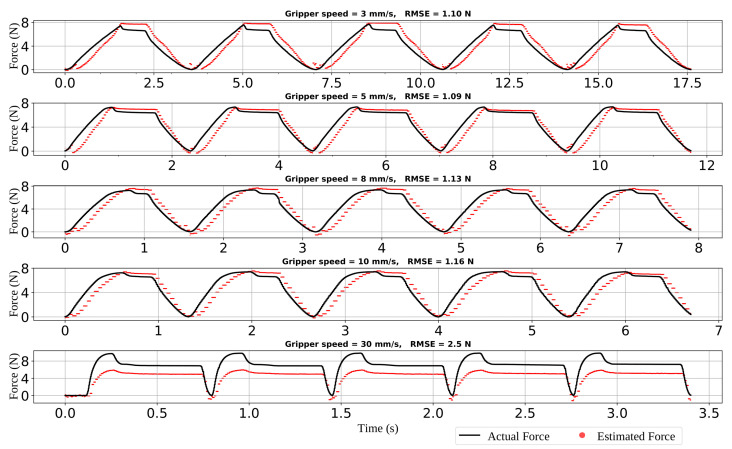
The bandwidth of the sensor: ground-truth force and estimated force at various frequencies. PhotoElasticFinger response was recorded at five different gripper velocities. At 30 mm/s, PhotoElasticFinger is failing to estimate the force.

Next, we evaluated the sensing performance at different squeezing velocities to validate the bandwidth of the sensor. Figure 7 illustrates the experiments performed to find the bandwidth of PhotoElasticFinger. The rubber fingertip was squeezed at five different velocities controlled by the gripper with five cycles.

From Figure 7, it can be observed that as the speed rises, the RMSE value increases, with the largest error of 2.5 N with the fastest gripping speed of 30 mm/s. At 5 mm/s and 3 mm/s velocities, the RMSE is around 1.1 N. At 10 mm/s, the RMSE is higher and equal to 1.16 N.

The bandwidth of our sensor is limited by both the sampling rate of the camera and the hysteresis (or retardation time) of the photoelastic silicone. Among these two limitations, the retardation time, which is present in the soft sensors, sets the main limit to the dynamic response of the photoelastic sensor. However, the durability and softness of the silicone-based sensors can outperform other sensing approaches, especially during precise manipulation.

We summarized the characteristics of the sensor in Table 1; it outlines the specification of the force sensor built upon the proposed photoelastic mechano-transduction method in comparison with the previously developed tactile sensors. The term “Softness” correspond to the compressibility of the sensor. The video of experimental grasping using the proposed sensor is available in the Supplementary Materials.

## 4. Conclusions

The proposed PhotoElasticFinger sensor can detect contact forces from 0.5 N to 8 N. The sensor uses a commodity camera to capture the changes of transmitted light through the photoelastic layer due to the polarization angle shift caused by the stress-induced birefringence. With the emerging soft robotics, where maintaining the proper contact between the robot and the environment is crucial for proper control, there is a fundamental need for soft rubber sensors. To enable soft robot—object interactions, we propose to use a hyperelastic silicone resin. Despite its obvious disadvantages, such as low bandwidth, the proposed sensor provides high sensitivity and proper grip control for the particular hardness of the resin. Moreover, in many robot—object manipulation scenarios, numerous try-and-error trials are necessary [44], demanding sensors robust to wearing off. The proposed in this work photoelastic sensor could be a wear-resistant solution thanks to the extra softness of the silicone resin.

Thus, in the proposed sensor, the displacement of the fingertip caused by the deformation of the soft pad is an important mediator that serves as a safe buffer between the environment and robot end-effector. On top of that, the methods used are computationally cheap, requiring only measuring the intensity of the transmitted light. In contrast, stiffer materials can provide faster response time (or lower retardation times), but their intrinsic dispersion results in more intricate patterns [30], demanding more sophisticated decoding methods, such as machine learning. As a result, it comes with a fair trade-off between the sensor’s dynamic response and processing times, which is typical for that type of sensor. To decrease the sensor’s response time but not lose its softness, it is possible to incorporate two or more different photoelastic materials into the same enclosure. By analyzing the more detailed intensity distribution maps using Neural Networks, a better response with the same hardware could potentially be achieved.

It should be noted that the approach described in this paper would fail to detect multiple contact points. This, however, could be mitigated by merging the photoelastic layer with multiple smaller fingertips and applying image processing techniques to analyze the regions of the silicone where intensity change occurs.

Besides the multiple contact point sensing, there is a need for detecting torsional and lateral forces with their applications in slip detection [9,45,46]. Indeed, the distributed light source, described in Section 2.3 can be used to detect the local changes in the refractive index, allowing a richer force analysis, such as analysis of the corresponding torsional and shear forces applied to the fingertip.

For future work, we plan to detect the tangential force and torsion that can be integrated in a sensory-motor control of a touch-driven robot arm by applying deep learning techniques, e.g., [47,48]. The silicone rubber used in our sensor is robust to tearing and wearing off and, therefore, the sensing characteristics of the sensor will not degrade from one set of repetitions, for example, grasping or sliding over surfaces, to another, which is beneficial for the aforementioned learning techniques, as it allows to collect larger data sets in training phases. 

## Figures and Tables

**Table 1:** Characteristics of the developed PhotoElasticFinger sensor in comparison with previously developed sensors. 1—Minimum resolvable normal force. 2—Dynamic range of normal force. 3—Softness (the maximum allowable displacement of the elastic sensing surface). 4—Non-linearity of force measurements. 5—Sampling Rate. 6—Bandwidth. 7—Sensing surface size.

	PhotoElasticFinger	Magnetic		Optical	Piezoelectric
		[42]	[10]	[25]	[43]
1	0.5 N	0.2 N	0.1 N	0.62 N	0.2 N
2	0.5–8 N	0.8 –5 N	0.1–5 N	0.62–8 N	0.2–2 N
3	5 mm	8 mm	1 mm	3 mm	1 mm
4	12%	-	-	9.5%	50%
5	approx. 20 Hz	1 kHz	0.04–2 kHz	20 Hz	230–800 fps
6	approx. 2 Hz	0.5 kHz	-	4 Hz	-
7	1.77 ${\mathrm{cm}}^{2}$	81 ${\mathrm{cm}}^{2}$	0.015 ${\mathrm{cm}}^{2}$	9.62 ${\mathrm{cm}}^{2}$	4.25 ${\mathrm{cm}}^{2}$

## Data Availability

The data presented in this study are available on request from the corresponding authors.

## Supplementary Materials

The following supporting information can be downloaded at: https://www.mdpi.com/article/10.3390/s22186807/s1, Video S1: Grasping using PhotoElasticFinger. Figure S1: Sensor Exploded view with the Gripper.

## Appendix A. Photoelastic Effect Description

### Appendix A.1. Photoelasticity Theory

The stress-induced birefringence can be described using a general stress-optical relation:

(A1) $$\Delta n_{ij}=-c_{ijkl}\sigma_{kl},$$

where $n_{ij}=n_{ij}-n_{0}I_{ij}$, $n_{0}$ is the refractive index of stress-free material, $n_{ij}$ is the refractive index tensor, $c_{ijkl}$ is the stress-optical tensor, $\sigma_{kl}$ is the stress tensor. By considering only two stress-optic coefficients $c_{1},c_{2}$ and due to the symmetry of refractive index tensor $n_{ij}$, stress tensor $\sigma_{kl}$ (and, therefore, $c_{ijkl}$), we can rewrite the stress-optical relation as:

(A2) $$\begin{array}{c} n_{x}=n_{0}-c_{1}\sigma_{x}-c_{2}\left(\sigma_{y}+\sigma_{z}\right)\\ n_{y}=n_{0}-c_{1}\sigma_{y}-c_{2}\left(\sigma_{x}+\sigma_{z}\right)\\ n_{z}=n_{0}-c_{1}\sigma_{z}-c_{2}\left(\sigma_{x}+\sigma_{y}\right), \end{array}$$

where $\sigma_{x}=\sigma_{11},\sigma_{y}=\sigma_{22},\sigma_{z}=\sigma_{33}$ are principal stresses, $n_{x}=n_{11},n_{y}=n_{22},n_{z}=n_{33}$ are principal indices of refraction. Therefore, the relative retardation (the angular phase shift $\Delta_{xy}$ for the polarization components) depends on the relative change in refraction index $n_{x}-n_{y}$ and, if we introduce the thickness of the layer *d*, can be derived as:

(A3) $$\Delta_{xy}=\frac{2\pi dC}{\lambda }\left(\sigma_{x}-\sigma_{y}\right),$$

where $C=c_{1}-c_{2}$ is the relative stress-optic coefficient, and λ is the wavelength. Such an angular phase shift results in the light intensity *E* change:

(A4) $$E=E_{0}\;sin^{2}\frac{\Delta_{xy}}{2}=sin^{2}\left[\frac{\pi dC}{\lambda }\left(\sigma_{x}-\sigma_{y}\right)\right],$$

where $E_{0}$ is the initial light intensity.

### Appendix A.2. The Details of the Numerical Simulation

First, to estimate the induced by applied force birefringence, a Structural Mechanics Module of the COMSOL Multiphysics^™^ was used to calculate the Von Mises stress distribution inside of a compressible material. The material’s mechanical properties (Sorta Clear 12, Smooth-on, Young’s modulus E=0.4MPa and Poisson’s ratio v≃0.38) were taken from the manufacturer’s website. The Neo-Hookean hyperelastic material model was used to calculate the stress–strain relation. To reduce the computational cost, a two-dimensional model was used to analyze the stress-strain behaviour. To apply pressure on the central hyperelastic disk (a photoelastic layer), two solid parts were displaced towards it with a step of 0.1 mm. At each step, a stationary direct solver MUMPS was used to calculate the stress $\bar{\bar{\sigma }}$ and strain $\bar{\bar{\epsilon }}$ tensors. Finally, the obtained stresses $\sigma_{x,y}$ were used to calculate the relative changes of refractive index $n_{x}-n_{y}$ using equations provided in Appendix A.1.

## Appendix B. Exploded View of the PhotoElasticFingertip

The PhotoelasticFinger (Figure A1) is fixed within the modular enclosure of the sensor using neodymium magnets, the location of which are shown in Figure A3. Cases for the polarizer, analyzer, and silicone were printed separately for the ease of assembly/disassembly. There are, in total, nine magnets. Five magnets hold polarizer and silicone cases, and the remaining four hold the analyzer and silicone cases, respectively. The diameter of each magnet is 6 mm, and the thickness is 1.3 mm, making the volumetric size 36.75 mm${}^{3}$ for each magnet.
