# Supplementary figures and images for: PhotoElasticFinger: Robot Tactile Fingertip Based on Photoelastic Effect
Source: Sensors (Basel). 2022 Sep 8;22(18):6807. doi: 10.3390/s22186807 (PMC9503177; doi:10.3390/s22186807)

Gripper

Clamping  
Magnet

Camera

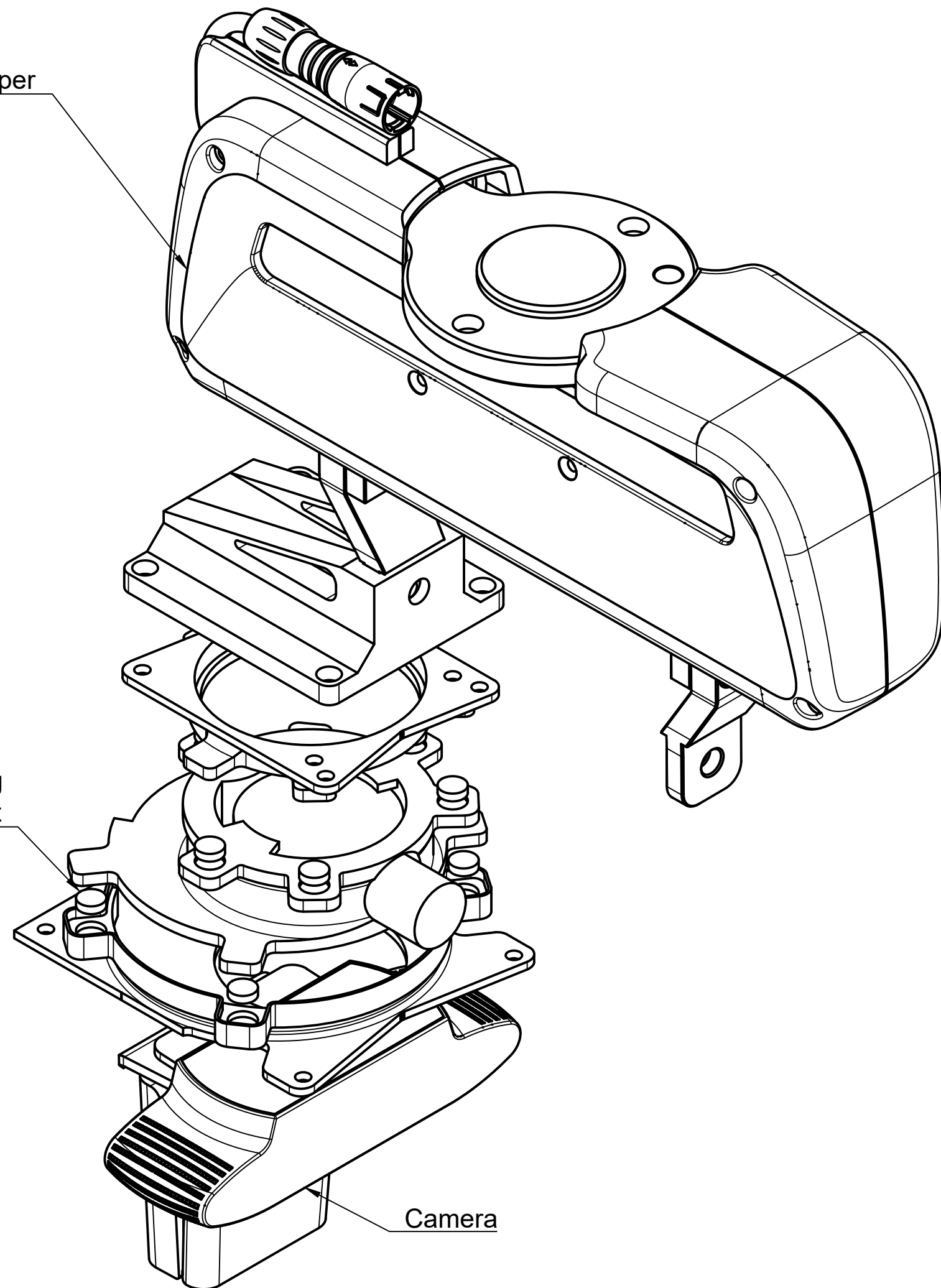

Supplement: Supplementary file 1 [file sensors-22-06807-s001.zip › Figure S1. Franka EE Drawing v1.pdf]
